# Graphene oxide enhances the specificity of the polymerase chain reaction by modifying primer-template matching

**DOI:** 10.1038/s41598-017-16836-x

**Published:** 2017-11-28

**Authors:** Yuanyuan Wang, Fengbang Wang, Hailin Wang, Maoyong Song

**Affiliations:** 1 State Key Laboratory of Environmental Chemistry and Ecotoxicology, Research Center for Eco-Environmental Sciences, Chinese Academy of Sciences, Beijing, 100085 P. R. China; 2 University of Chinese Academy of Sciences, Beijing, 100049 China; 3 Institute of Environment and Health, Jianghan University, Wuhan, 430056 P.R. China

## Abstract

Aiming at improved specificity, nanoparticle assisted polymerase chain reaction (PCR) has been widely studied and shown to improve PCR. However, the reliability and mechanism of this method are still controversial. Here, we demonstrated that 1 μg/mL of graphene oxide (GO) effectively enhances the specificity of the error-prone multi-round PCR. Mismatched primers were designed as interference to produce nonspecific products when the same amounts of matched and mismatched primers were added into semi-multiplex PCR. It was found that GO can enhance specificity by suppressing the amplification of mismatched primers. We monitored the primer-template-polymerase-GO interactions involved in the PCR using a capillary electrophoresis/laser-induced fluorescence polarization (CE-LIFP) assay. The results showed that the addition of GO promoted the formation of a matched primer-template complex, but suppressed the formation of a mismatched primer-template complex during PCR, suggesting that interactions between the primers and GO play an essential role. Furthermore, we successfully amplified the FOXL2 gene from PEGFP-N1 vectors using GO to eliminate the nonspecific products in PCR. Taken together, these results suggest that the GO can be used as an efficient additive for improving the conventional PCR system.

## Introduction

Polymerase chain reaction (PCR) amplifies a specific region of a DNA strand to generate thousands to millions of copies of a particular DNA sequence. PCR has become one of the most important techniques in modern biological and medical science. It has a variety of applications, including DNA cloning for sequencing, functional analysis of genes, diagnosis of hereditary and infectious diseases, and identification of genetic fingerprints^1–10^. However, PCR is not always specific. Nonspecific DNA fragments are often produced, especially in both multiple-round and multiplex PCR^11^. As a result, a variety of additives have been employed to enhance the specificity of the PCR, including single-stranded DNA-binding proteins (SSBs)^12^, betaine^13^, tetramethylammonium chloride (TMAC)^14^, and TMAC derivatives^15^. Due to the limitations of conventional methods, specificity in PCR amplification still remains a challenge, even with sophisticated optimization.

Gold nanoparticles and reduced graphene oxide (RGO) were recently reported to reduce nonspecific fragment formation in multiple-round PCR^16–18^. This enhancement effect of nanoparticles on PCR specificity might be attributable to two potential mechanisms: selective binding to single-stranded DNA (ssDNA) in a manner analogous to SSB16 and heat transfer enhancement by the superior energy transport properties of nanoparticles^17,19^. However, a contradictory report found that the gold nanoparticles did not increase specificity, but instead favoured smaller products over larger products, regardless of specificity^20^. These contradictory reports show that the evidence is inconclusive as to whether nanoparticles are efficient additives for improving PCR specificity.

In this work, we systematically investigated the ability of graphene oxide (GO) to enhance PCR specificity. We found that GO effectively enhanced the specificity of error-prone two-round PCR. Using designed mismatched primers as interference, we demonstrated that matched primers were preferable for use in PCR in the presence of GO. We further confirmed that GO promoted matched primer-template complex formation and suppressed mismatched primer-template complex formation during PCR using a capillary electrophoresis/laser-induced fluorescence polarization (CE-LIFP) assay.

## Results and Discussion

### Characterization of GO

**Figure 1:**
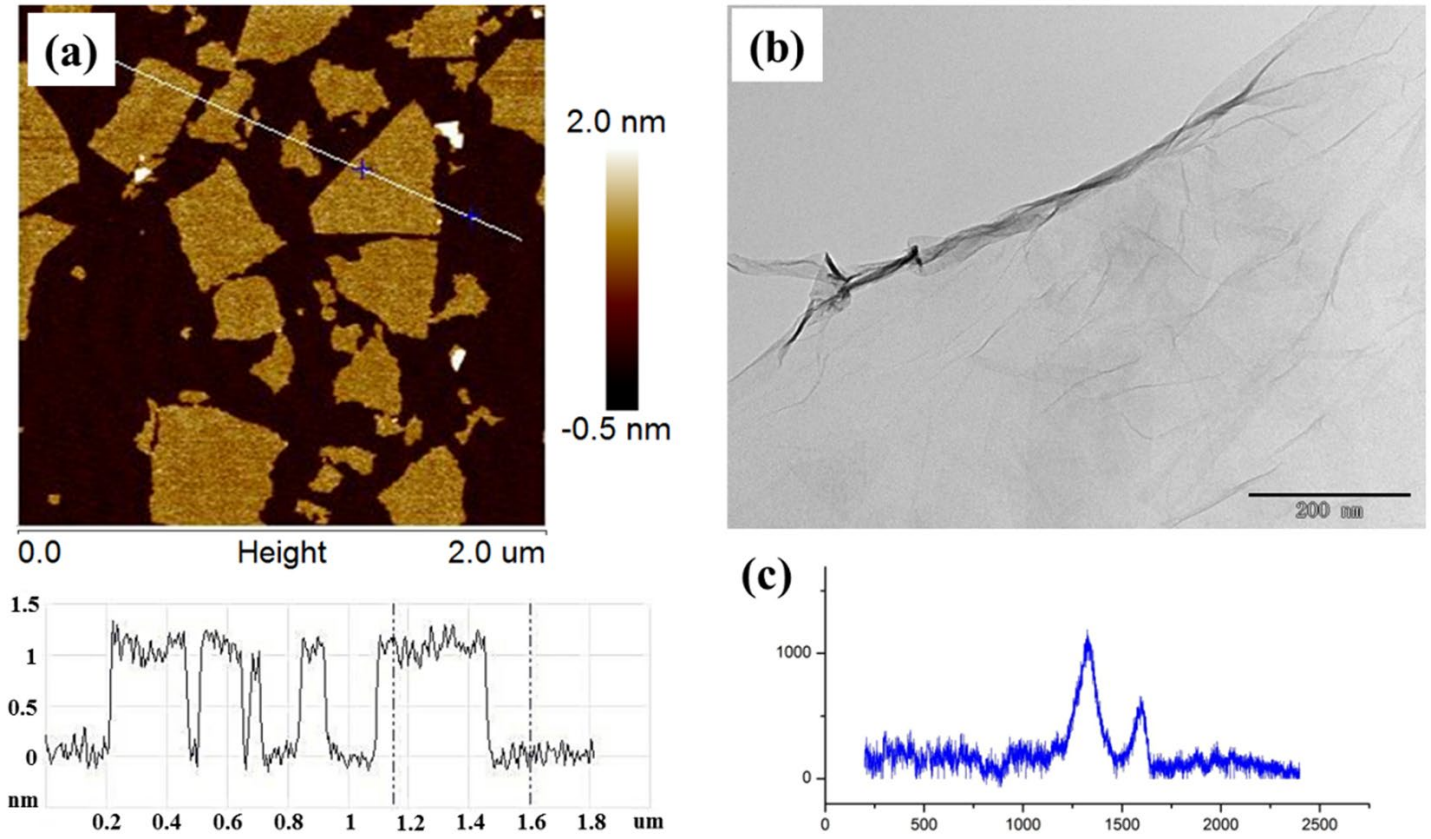
Characterization of GO: AFM (**a**), TEM (**b**), and Raman spectra (**c**).

As shown in Fig. 1a, the GO nanosheets exhibited a thickness of ∼1.0 nm and a size of 0.2–0.5 μm in the AFM image. Independent GO sheets were observed in the TEM image (Fig. 1b). Dark areas indicated the thick stacking nanostructure of several GO layers with the presence of oxygen functional groups. Areas of higher transparency showed thinner films with fewer GO layers, resulting from stacking nanostructure exfoliation. In the Raman spectrum of the GO, two peaks were visible corresponding to the G band at 1600 cm^−1^, derived from the graphite structure, and the D band at 1345 cm^−1^, derived from defects (Fig. 1c).

### GO-assisted multi-round PCR

**Figure 2:**
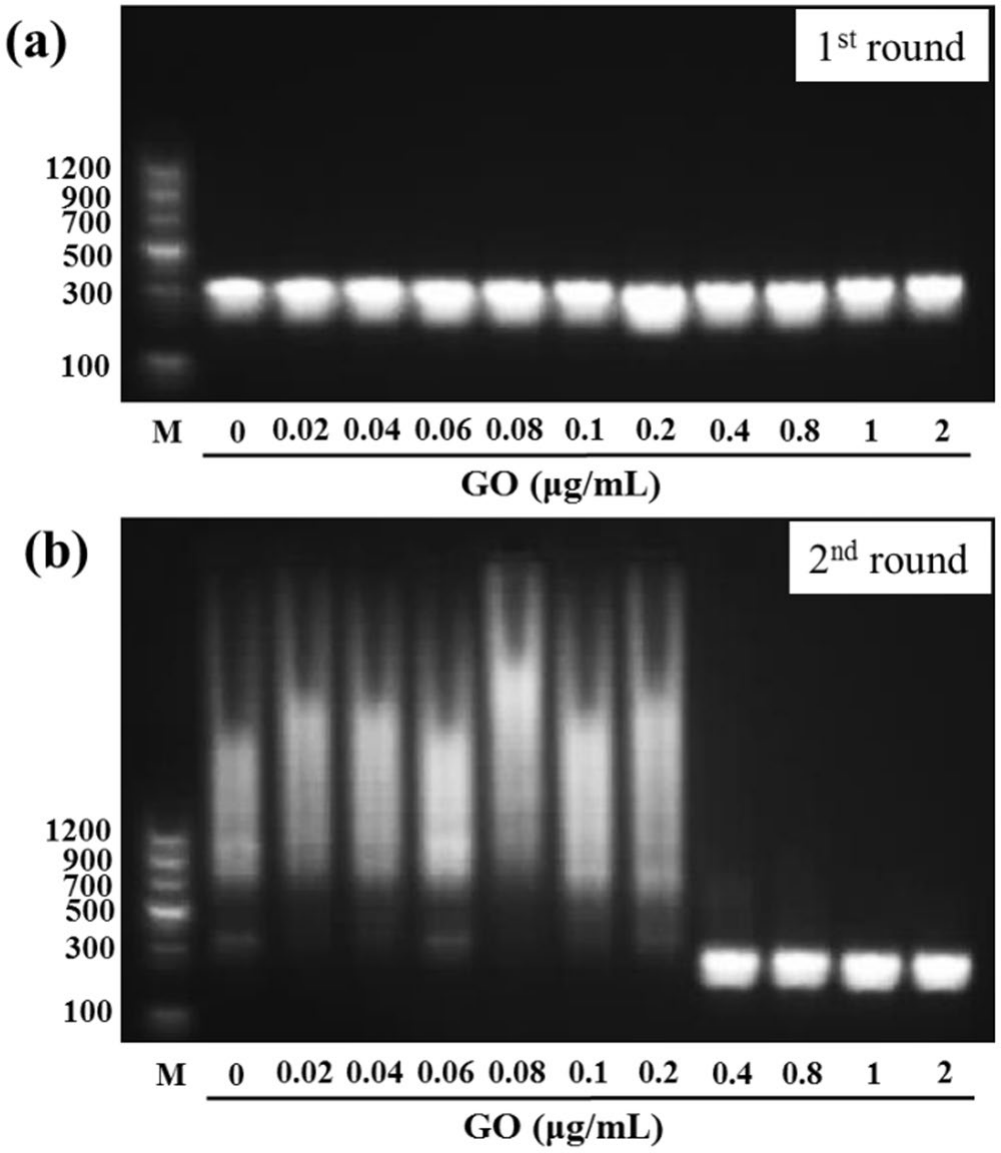
The effect of GO on first-round (**a**) and second-round (**b**) PCR. PCR was performed by employing a 283 bp target sequence from Lambda DNA template, and PCR products were analyzed by agarose gel electrophoresis (1.5%). Lane M is markers.

We employed an error-prone multi-round PCR to investigate the effect of GO on DNA amplification^25,26^. Here, a pair of primers (forward primer and reverse primer R1) was used to amplify a 283-bp target sequence from lambda DNA. As shown in Fig. 2, in the absence of GO, when the PCR product was employed as the template in the second round of PCR, nonspecific PCR products were observed, as demonstrated by a broad molecular size distribution of amplified products in agarose gel electrophoresis. Strikingly, in the presence of GO, the non-specific bands disappeared, and only a single 283-bp band was observed with increasing GO concentration. These results indicate that GO can enhance the specificity of two-round PCR. Target sequence amplification was uninhibited when the concentration of GO was lower than 2 μg/mL. However, when excessive GO (>4 μg/mL) was added to the PCR mixture, the amplification was inhibited in both first and second round PCR (data not shown), indicating that the concentration of GO is critical for obtaining optimal PCR results. In fact, we could observe the target band even after the 5th round of PCR with the elimination of nonspecific products by 1 μg/mL of GO (data not shown).

### Suppression of mismatched primers by GO

**Figure 3:**
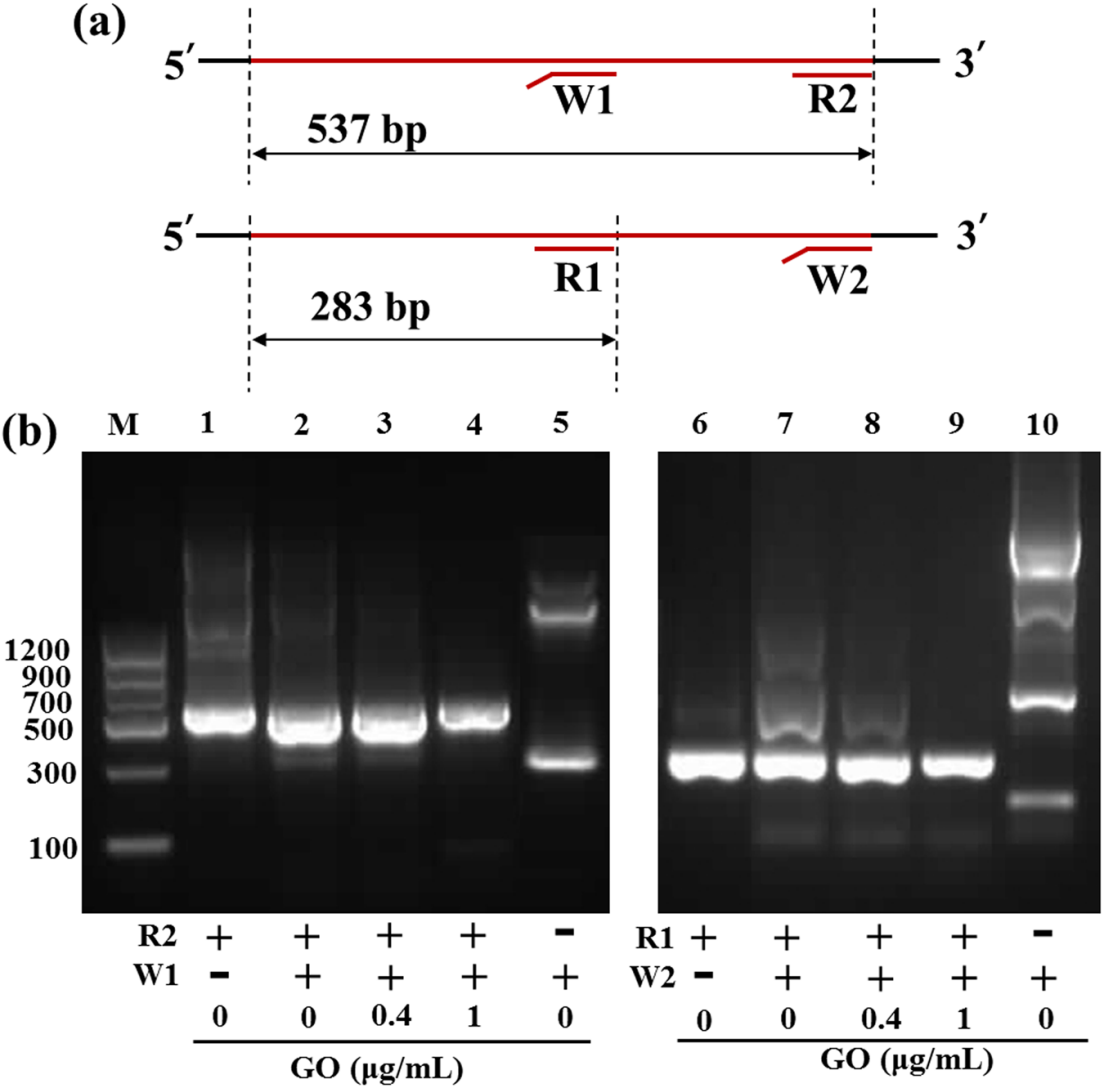
(**a**) Illustration of semimultiplex PCR with a forward primer and two reverse primers (R1 + W2 and R2 + W1). (**b**) The effect of GO on DNA amplification in a semimultiplex PCR. Target sequences for R1 and R2 were 283 and 537 bp, respectively. The final concentrations of R1, W1, R2 and W2 were 20 nM. PCR products were analyzed by agarose gel electrophoresis (1.5%). Lane M is markers.

Specificity of primer-based amplification reactions depends on the specificity of primer hybridization and extension. Here, two designed reverse primers (see Table 1), which contained mismatched bases, were introduced into the reaction mixture as contaminates to produce nonspecific products (Fig. 3a). As shown in Fig. 3b, the product primer by match primer R1 only was a 283-bp target band (lane 6), while the products primed by mismatch primer W2 resulted in several nonspecific bands and a DNA smear (lane 10). When R1 and W1 were used as primers together in the semi-multiplex PCR assay, the major PCR product was a 283-bp target band, but the nonspecific products still appeared in the agarose gel electrophoresis due to W2 contamination (lane 7). Annealing temperature has a strong influence on PCR specificity, but these nonspecific products cannot be completely eliminated by optimizing the annealing temperature (data not shown). However, only a target band was observed, and the nonspecific products disappeared when the concentration of added GO was 1 μg/mL (lane 9), indicating that GO suppressed the formation of nonspecific products with the mismatch-containing primer W2. A similar phenomenon was also observed in a semi-multiplex PCR assay with two different reverse primer pairs (R2 and W1) (lane 1–5), indicating that GO can enhance the specificity of PCR by suppressing the mismatched primers. Interestingly, GO could enhance the specificity of PCR with the perfect-matched primer R2 only (lanes 1 and 4).

**Table 1:** Primers for Lambda and FOXL2 templates.

Template	Name*	Sequence (5′–3′)	Amplicon length
Lambda DNA	FP	GGCTTCGGTCCCTTCTGT	
	R1/F-R1	CACCACCTGTTCAAACTCTGC	283 bp
	W1/F-W1	CACCACCTGTTCAAACTC**ACG**	283 bp
	R2	GTTAGAAACCGACAGCGTG	587 bp
	W2	GTTAGAAACCGACAGC**AGC**	587 bp
FOXL2 gene	FP	TGTCATGATGGCCAGCTACCCCG	1130 bp
	R	CTCTCAGAGATCGAGGCGCGAATG	

*In primer names, FP indicates the forward primer. R and W indicate matched reverse primer and mismatched reverse primer, respectively. The letter of “F-” before the primer name indicates that primer was labeled with TAMRA fluorochrome at 5′ end.

**Figure 4:**
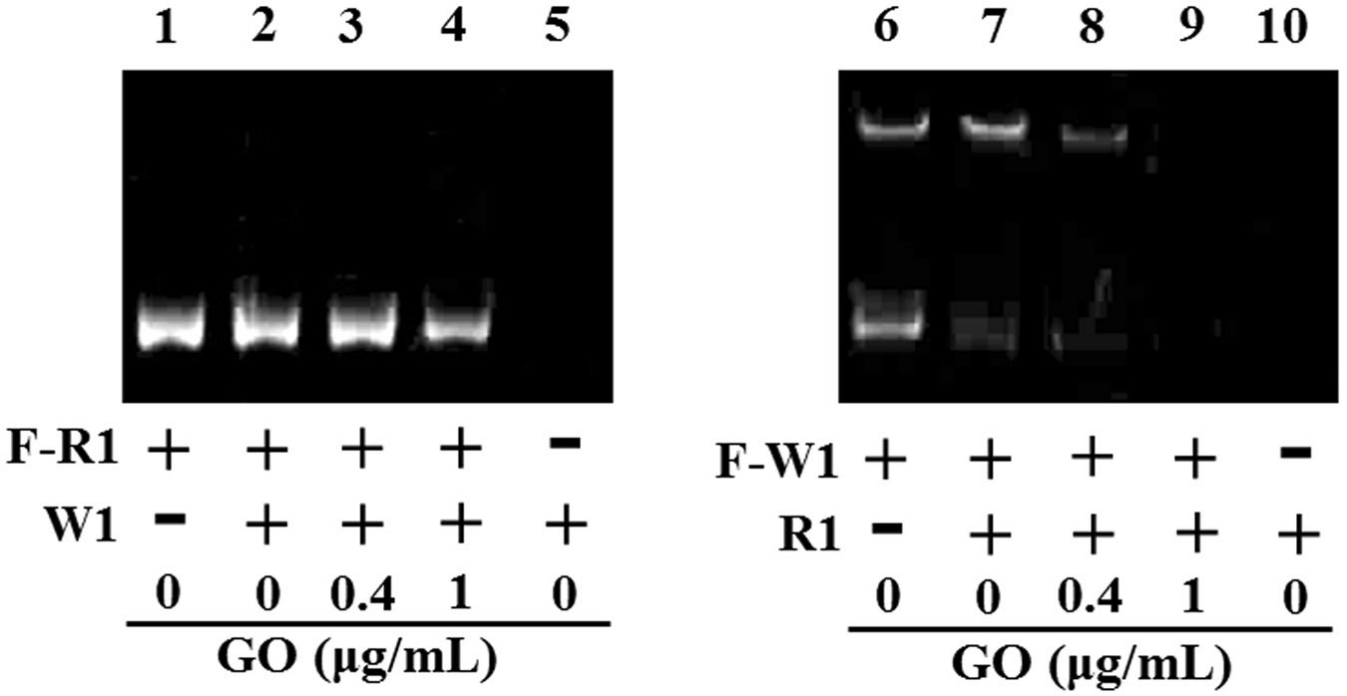
The effect of GO on DNA amplification in a semimultiplex PCR with a forward primer and two reverse primers (F-R1 + W1 and R1 + F-W1). F-R1 and F-W1 were labeled with TAMRA fluorochrome at 5′ end. The final concentrations of R1 and W1 were 20 nM. PCR products were analyzed by agarose gel electrophoresis (1.5%). Lane M is markers.

As demonstrated in Fig. 3a, primers R1 and W2 (or R2 and W1) were bound to different sites of the template, indicating that the amplification of specific and nonspecific products was non-competitive. However, primer W1 had the same 18 bases as primer R1 at the 5′ ends, indicating that W1 can competitively bind to the R1 binding site on the DNA template. The 283-bp band in the PCR products of W1 had a similar size as that of R1 (Fig. 2b, lanes 5 and 6). Due to the exonuclease activity of polymerase, this “target” sequence may be right copies of template. However, it is neither economic nor convenient to check the specificity of each PCR product using DNA sequencing. To further investigate the effect of GO on PCR specificity, a semi-multiplex PCR assay was carried out with two pairs of site-competitive primers (F-R1 + W1 and R1 + F-W1). Here, we used the fluorescence labelled (5′-TAMRA) primers to identify the origin of the PCR products. As shown in Fig. 4, lane 2, only a single band appeared in the gel using F-R1 + W1 as primers. Comparing this to primer F-R1 (lane 1) or W1 only (lane 5), this visible band came from the PCR product of F-R1. However, two bands appeared in the gel using F-W1 + R1 as primers (lane 7), and these bands came from the PCR product of F-W1. As the GO concentration was 1 μg/mL, visible bands formed as F-W1 disappeared (lane 9), but the band formed by F-R1 (lane 4) was still observed in the gel. This result suggests that the amplicons formed by mismatch primer F-W1/W1 were completely suppressed by the addition of GO.

### Mechanism of GO-assisted PCR

Recently, Huang *et al*. reported the interaction between GO surface and *Pfu* DNA polymerase could affect the specificity of PCR^27^. Here the interaction of the polymerase and GO was investigated by the fluorescence quenching experiments (Figure S1). The fluorescence intensity of polymerase decreased with increasing concentration of GO and the maximum emission wavelength was slightly red shifted, indicating that the microenvironment of fluorophores in polymerase was changed after addition of GO. In our previous study, pristine fullerene nanoparticles are capable of adsorbing polymerase and significantly inhibiting its biologically important replication activity^28^. Here we used real-time PCR to measure the activity of polymerase in the presence of GO. As shown in Figure S2, GO could enhance the amplification efficiency of polymerase at concentrations lower than 1 μg/mL, but inhibit the activity of polymerase at higher concentrations (>2 μg/mL) whatever using matched or mismatched primers. We presumed that presence of GO increased the amplification rate by protecting polymerase from inactivation, which was similar to the protective effect of GO on horseradish peroxidase^29^. The results also indicated that GO did not enhance the specificity of polymerase because the PCR product was not supressed by addition of GO when only using mismatched primers (Figure S2b).

GO can preferentially bind to ssDNA through *π–π* stacking interaction and hydrogen bonding^30^, whereas double-stranded DNA (dsDNA) has a weak affinity for the GO surface due to hidden nucleobases inside the double helix^31,32^. Due to this selectivity, GO can be used for the sequence-specific detection of DNA^33,34^, and the adsorbed ssDNA can be released from the GO by forming a double helix with its target sequence^31^. During DNA amplification in PCR, the dsDNA template was first denatured to two ssDNA molecules by heating it to 94–98 °C at the start of each cycle. In the next step, the primers were bound with each of the ssDNA templates while the temperature was lowered to 50–60 °C. Here, we observed that the addition of GO suppressed the amplification with mismatched primers. Thus, we hypothesize that this specificity arises as a result of the interactions between the primers, template, and GO during PCR. To test this hypothesis, we analysed the interactions between dye-labelled primers with a template or polymerase and with or without addition of GO in a simulative semi-multiplex PCR (with no dNTPs).

Here we firstly compared the formation of matched and mismatched primer-template complexes in the present of GO. The strong interaction between ssDNA and GO facilitated the fluorescence quenching of the fluorophore by GO. In the presence of a complementary target DNA, the binding between primer and template will disturb the interaction between primers and GO, and release ssDNA from GO, resulting in restoration of fluorophore fluorescence^33,34^. As shown in Figure S3, in the presence of GO, nearly 100% quenching of the fluorescence for both TAMRA labelled matched and mismatched primer was observed. A significant enhancement in fluorescence was observed after addition of template to primer-GO. This indicated that not only matched primer (Figure S3a) but also mismatched primer (Figure S3b) can hybridize with template and release them from GO resulting in restoration of fluorescence. We employed the fluorescence restoration rate to reflect the affinity between matched or mismatched primer with the template. As shown in Figure S4, matched primer showed a higher fluorescence restoration rate than that of mismatched primer. This comparison suggested that matched primer was easier to form primer-template complex than mismatched primer in the presence of GO. However, it should be further investigated whether it worked by addition of a low concentration GO in Simulative semimultiplex PCR.

**Figure 5:**
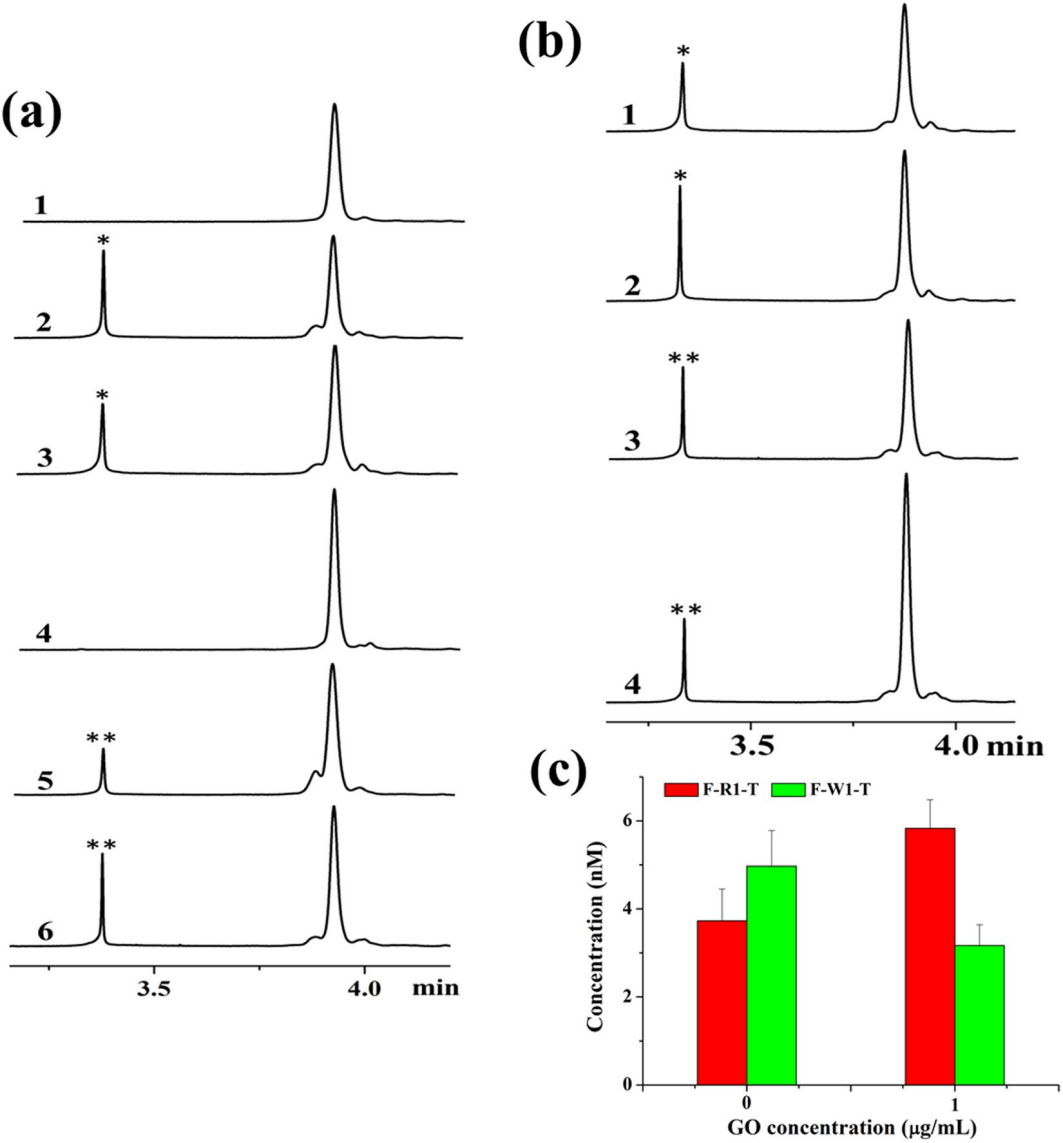
(**a**) CE-LIFP analysis of the interaction of F-R1 and F-W1 with template and polymerase. Reaction solutions contained 1: F-R1; 2: F-R1 and template; 3: F-R1, template, and polymerase; 4: F-W1; 5: F-W1 and template; 6: F-W1, template, and polymerase. (**b**) CE-LIFP analysis of complex conformation in the presence of GO. Reaction solutions contained 1: F-R1, template, and polymerase; 2: F-R1, template, polymerase, and GO; 3: F-W1, template, and polymerase; 4: F-W1, template, polymerase, and GO. (**c**) Change of F-R1-template and F-W1-template complex concentrations in the presence of GO. The concentrations of primers (F-R1 and F-W1), template, and polymerase are 10 nM, 10 nM, and 0.5 U, and the concentration of GO is 1 μg/mL. All reaction solutions were run with a cycle of 45 s at 95 °C, 1 min at 50 °C, and 1 min extension at 72 °C before CE-LIFP analysis.

In our previous studies, we demonstrated the use of CE/LIFP for dynamic monitoring of DNA-DNA, DNA–protein and protein–protein interactions^21–24^. Here, we monitored the primer–template–polymerase–GO interactions involved in PCR using CE/LIFP assays. The results of the fluorescence polarization obtained from the CE/LIFP assays are summarized in Table 2. Three detectable complexes, including primer-template, primer-polymerase, and primer-template-polymerase, should have formed in the solution in the absence of GO. As shown in Fig. 5a, only the primer-template complex was observed during the CE process through measuring both the mobility and fluorescence polarization of the complex simultaneously. The increase in fluorescence polarization reflected the increase in the molecular size when primers were bound to the templates. In the semi-multiplex PCR, both the F-W1–template complex and the F-R1–template complex were formed in both the presence and absence of GO (Fig. 5b). However, the peak of F-R1–template complex increased, while that of F-W1–template complex decreased in the presence of 1 μg/mL of GO. The quantification of each complex showed that the concentration of the F-R1–template complex increased from 3.73 to 5.83 nM, while the concentration of the F-W1–template complex decreased from 4.97 to 3.17 nM, simultaneously (Fig. 5C). We performed the experiments by changing the cycles and template concentrations, and similar phenomena were observed (data not shown). This indicates that GO can suppress the formation of the mismatched primer-template complex in each PCR cycle.

**Table 2:** Fluorescence polarization of each peak obtained from CE-LIFP.

Reaction system	Fluorescence polarization (*P* values)		
	Peak*	Peak**	Peak***
F-R1	ND	0.147	ND
F-R1 + T	0.258	0.146	ND
F-R1 + T + P	0.255	0.147	ND
F-R1 + T + P + GO	0.256	0.148	ND
F-W1	ND	0.148	ND
F-W1 + T	0.167	0.142	ND
F-W1 + T + P	0.169	0.142	ND
F-W1 + T + P + GO	0.166	0.144	ND

T: template; P: polymerase; ND: not detected.

Based on the data described in section 3.3, R1 suppressed the amplification by F-W1 (Fig. 4, lanes 6 and 7), but W1 showed no obvious suppression of the amplification by F-R1 (Fig. 4, lanes 6 and 7) in the absence of GO. It suggests that polymerase tends to use match primer preferentially due to its specific recognition of primer-template complex in the semimultiplex PCR (Figure S5). In the presence of GO, it can improve match primer-template complex formation. The absolute suppression of nonspecific product formed with W1/F-W1 (Fig. 4, lanes 4 and 9) suggests that the proportion change between R1–template complex and W1–template complex may play an important role in enhancing PCR specificity. We further compared the effect of GO on PCR specificity using a series nonspecific primers. These primers have different binding affinity to template by containing different numbers of correct template-binding bases (18 to 24 bases). As competitive primers to R1, these primers can produce nonspecific PCR products as well as W1. Similarly, the amplification of nonspecific PCR products with these mismatch primers also can be inhibited by addition of GO at optimized concentrations (data not shown).

**Figure 6:**
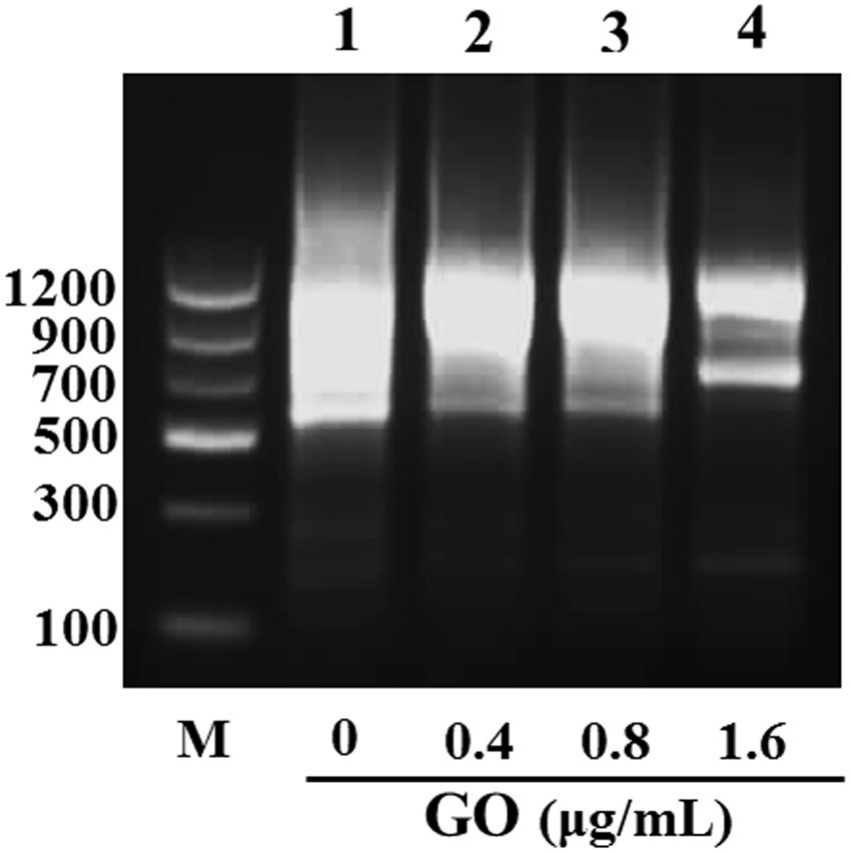
The effect of GO on FOXL2 gene amplification. The coding sequence of FOXL2 gene cloned to the PEGFP-N1 vector was used as the template. PCR was performed by employing an 1130 bp target sequence from PEGFP-N1 vector template. PCR products were analyzed by agarose gel electrophoresis (1.5%). Lane M is markers.

Numerous studies have reported that nanomaterials, including gold nanoparticles and Titanium dioxide (TiO_2_) nanoparticles, enhanced amplification of GC-rich PCR^26,35–37^. Here we used a GC-Rich PCR system with low specificity to further confirm the effect of GO on PCR specificity. The coding sequence of the FOXL2 gene cloned to the PEGFP-N1 vector was used as the template. A pair of primers with a high GC content of 72% (Table 1) was used to amplify the 1130-bp FOXL2 gene from the PEGFP-N1 vector. As shown in Fig. 6, in the absence of GO, nonspecific PCR products appeared as a broad molecular size distribution of amplified products in the agarose gel electrophoresis (Lane 1). However, two separate bands were observed when GO was added at 1.6 μg/mL (Lane 4). The sequencing results of the 1130-bp PCR products demonstrated that the addition of GO did not interrupt the fidelity of the PCR. Interestingly, Taq polymerase cannot amplify the FOXL2 genes from the PEGFP-N1 vector, even in the presence of GO, indicating the mechanism of GO-assisted PCR remains to be explored.

To allow the extensive use of molecular methods in medical practice, scientific research is nowadays strongly focusing on the fully integrated technological solutions for nucleic acids analysis^38^. In these systems, PCR is one of the most fundamental techniques to amplify low-copy DNA in modern biological and medical sciences^17,36^. Nonetheless, further improvements of the existing protocols are required to broaden the applicability of PCR for routine diagnostic purposes, to enhance the specificity and the yield of PCRs as well as to reduce the costs for high-throughput applications^39^. Recently, the applications of nanomaterial-assisted PCR (nanoPCR) have received considerable attention. In this study, we demonstrated that GO could improve the performance of PCR by enhancing its specificity at a low concentration.

Moreover, with a large lateral size, rich chemical, optical, and mechanical properties, GO performed positively in many areas of biological analysis. Min *et al*. developed a new GO-based platform for Endonucleases (ENase) and methyltransferase (MTase) activity assays. This strategy can be further employed in a DNA MTase activity assay in which the DNA strands methylated by MTases cannot be hydrolyzed by ENases. GO was used to perform ATP live-cell imaging, which can semi-quantified ATP in live cells using nonspecific desorption of DNA from GO as the internal reference^40^. Yang *et al*. demonstrated that GO can effectively protect RNA probes from enzymatic digestion^41^. This finding offers an exciting new way to stabilize ssRNA probes for analysis of nucleic acids, proteins, and small molecules. Lin *et al*. designed an aptamer-carboxyfluorescein (FAM)/graphene oxide nanosheet (GO-nS) nanocomplex to investigate its ability for molecular probing in living cells^42^. They found that GO could be a robust candidate for many biological fields, such as DNA and protein analysis, gene and drug delivering, and intracellular tracking. Graphene platform was employed to combine the sensitivity of electrochemical impedance spectroscopy with the high selectivity of hairpin-shaped DNA probes for the rapid detection of single nucleotide polymorphism correlated to the development of Alzheimer’s disease^43^.

## Conclusions

We found that GO significantly improved PCR specificity at appropriate concentrations. We demonstrated that in the presence of GO, the nonspecific products formed by mismatch primers can be suppressed in PCR. We also showed that interactions between GO and the DNA template or primers may enhance PCR specificity. Addition of GO enhances the formation of the matched primer-template complex but suppresses that of the mismatched primer-template complex while favouring PCR specificity. Our results demonstrated that GO has great potential for use as a PCR enhancer.

## Materials and Methods

### Characterization of Graphene Oxide

Graphene oxide dispersion was obtained from Nanjing XFNANO Nanomaterials Technology Co., LTD (China). It was sonicated in a water bath (KQ-300DB, 40 kHz) for 10 min before use. GO dispersion was diluted to 2 µg/mL, after which 3 μL of the diluent was deposited on a copper grid and dried overnight in a clean box. The GO morphology and structure were imaged with an H-7500 transmission electron microscope (TEM, Hitachi, Tokyo, Japan). This nanomaterial was also characterized by a multimode atomic force microscope (AFM, Bruker, Germany) in ScanAsyst mode. In addition, Raman characterization of the GO was performed using an inVia Raman spectroscope (Horiba, Japan) with a laser source at 780 nm and 25 mW.

### DNA templated and primers

The DNA templates used were lambda DNA (Promega, USA) and PEGFP-N1 vectors containing the coding sequence of the FOXL2 gene provided by Professor Tang of Weifang Medical University. The primers for the lambda DNA and FOXL2 gene are listed in Table 1, and the mismatched bases are underlined. The letter “F” before the primer name indicates that primer was labelled with a TAMRA fluorochrome at the 5′ end. The primers were synthesized by Shanghai Sangon Bio-technology Co. (China).

### PCR amplification and products analysis

The conventional PCR reaction was carried out with 6 ng of DNA template, 0.5 µM of each primer and 10 µL of GoTaq green master mix (Promega) containing buffer, nucleotides, and Taq polymerase in a final volume of 20 µL. PCR was run with 35 cycles of 45 s denaturation at 95 °C, 1 min of annealing at 50 °C, followed by a 1 min extension at 72 °C. The PCR procedure for the FOXL2 gene was 35 cycles of 30 s denaturation at 94 °C, 30 s of annealing at 64 °C and a 1 min extension at 72 °C. Cycling was started after an initial denaturation at 95 °C for 3 min and ended with a final extension at 72 °C for 5 min. The polymerase used for FOXL2 gene amplification was LA taq DNA polymerase (TaKaRa Bio.Inc.) All amplifications were carried out in the MycyclerThermal Cycler system (Bio-Rad Inc.). Semi-multiplex PCR was performed using two reverse primers of the same concentration (the concentration of each primer was 0.5 µM). In this study, three pairs of reverse primers, including R1 + W1, R1 + W2, R2 + W1, were used. All experiments were performed in triplicate.

PCR products were analysed by 1.5% agarose gel electrophoresis using a horizontal electrophoresis instrument (Baygene, China). Gels were stained with ethidium bromide unless otherwise stated, visualized on a UV transilluminator, and photographed by the gel imaging system (UVP, USA). PCR amplicons of the target region in the FOXL2 gene was sequenced by Sanger sequencing (Sangon, China).

### Simulative semimultiplex PCR

The interactions of primer–template–polymerase–GO were performed in a simulative semi-multiplex PCR. Two TAMRA labelled primers, F-R1 and F-W1, were used for detecting fluorescence. A designed 80-bp ssDNA was used as the template instead of lambda DNA. Its sequence was GTCAGTATGCTGCGTGTTGAGTTCAGC**GCAGAGTTTGAACAGGTGGT**GAACTGATGCAGGATATCCGGCAGGAAACACTG. The underlined section is complementary to primer R1. F-R1 (20 nM), W1 (20 nM), and Taq polymerase (0.5 U) were added to 20 μL 1 × TH buffer (20 mM Tris-HCL buffer, pH 7.4, 10 mM Mg^2+^). To simulate different template concentrations during PCR, the concentration of the added template ranged from 0.2 to 20 nM. Another pair of primers, F-W1 + R1, were used in a parallel experiment. The mixture was run with a cycle of 45 s at 95 °C, 1 min at 50 °C, and a 1 min extension at 72 °C. The samples were then subjected to CE-LIFP analysis. Similarly, the effects of the GO were studied according to the above methods by adding 1 μg/mL of the GO to the simulative semi-multiplex PCR. All experiments were performed in triplicate.

### CE-LIFP analysis

CE-LIFP analysis was conducted on a laboratory-built CE-LIFP system^21–24^. The fused-silica capillary (30 cm × 25 μm i.d.) used for separation was obtained from Yongnian Optic Fibre Plant (Hebei, China). Briefly, the capillary was systematically flushed for 1 min with 0.02 M NaOH, 3 min with deionized water and then with running buffer (1 × TG buffer, pH 8.3) for 5 min before each analysis. A positive voltage of 15 kV was applied for the electrokinetic injection, and 20 kV for the CE separation. The fluorescence was split into the horizontally and vertically polarized beams by a polarizing beam splitter (Melles Griot, Nepean, Canada) and was detected by two photomultiplier tubes (PMT, model R1477, Hamamatsu Photonics, Japan) at 575 nm. The fluorescence polarization (FP) values were calculated from the intensity of both the horizontally (*I*_*h*_) and vertically (*I*_*v*_) polarized fluorescence values according to the following equation:

$$P=\frac{{I}_{v}-{I}_{h}}{{I}_{v}+{I}_{h}}$$

## Electronic supplementary material

SI
