# Supplementary material for: Graphene oxide enhances the specificity of the polymerase chain reaction by modifying primer-template matching
Source: Sci Rep. 2017 Nov 28;7:16510. doi: 10.1038/s41598-017-16836-x (PMC5705716; doi:10.1038/s41598-017-16836-x)
Supplement: Supplementary file 1 — SI [file 41598_2017_16836_MOESM1_ESM.pdf]

Graphene oxide enhances the specificity of the polymerase chain reaction by modifying primer-template matching

Yuanyuan Wang[1,2], Fengbang Wang[1,2], Hailin Wang[1], Maoyong Song[1,3]*

[1]State Key Laboratory of Environmental Chemistry and Ecotoxicology, Research Center for Eco-Environmental Sciences, Chinese Academy of Sciences, Beijing 100085, PR China

[2]University of Chinese Academy of Sciences, Beijing 100049, China

[3]Institute of Environment and Health, Jianghan University, Wuhan 430056, P. R. China

*E-mail: smsong@rcees.ac.cn. Tel/Fax: 0086-10-62923597.

Methods

Fluorescence spectra of polymerase. Fluorescence emission spectra were recorded in the range of 300–450 nm on a LS-55 spectrofluorimeter (PE, USA) equipped with 100 μL quartz cells. The bandwidth of excitation and emission (3 nm) and scan speed (300 nm/min) were kept constant within each data set. All measurements were performed at room temperature (~25 °C). All experiments were performed in triplicate.

Real-time PCR. The real-time analysis of the template DNA amplification was performed using a Mx3005P QPCR Systems (Stratagene, USA). Brilliant SYBR QPCR Master Mix kit (Stratagene, USA) was used as the real-time PCR reagents. This 2× master mix contained all of the reaction components except the template and primers. All the reagents were dispensed into a 0.2 mL 8-strip tube with a ultimate volume of 20 μL, containing 8 μL of 2× master mix, 1μL of 10 nM template, 0.5 μL of 10 μM forward primer, 0.5 μL of 10 μM reverse primer, and 10 μL of GO solution. The subsequent temperature cycling program include 94 °C for 45 s, 55 °C for 1 min, and 72 °C for 1 min, 40 cycles, and final extension at 72 °C for 10 min. A positive control (normal RT-PCR without GO solution) was included with each set of PCR experiments.

The sequence of ssDNA template (80 bases) is 5'-GTCAGTATGCTGCGTGTTGAGTTCAGCGCAGAGTTTGAACAGGTGGTGAACTGATGCAGGATATCCGGCAGGAAACACTG-3', which was synthesized by Sangon Biological Engineering Technology and Services (Shanghai, China). The sequence of forward primer is 5'-GGCTTCGGTCCCTTCTGT-3', the sequence of matched reverse primer is 5'-CACCACCTGTTCAAACTCTGC-3', and the sequence of matched reverse primer is 5'-CACCACCTGTTCAAACTCACG-3'.

Primer-template interacion assay. TAMRA labelled ssDNA (matched and mismatched primer, 10 nM) was mixed with GO (20 mg/L) for 5 min prior to the addition of target DNA (template). The final template concentration in samples ranged from 0 to 100 nM. After allowing this mixture to stand for about 30 min at room temperature, the fluorescence of the mixture was detected. The TAMRA was excited at 542 nm, and fluorescence intensity at 568 nm was measured using a LS-55 spectrofluorimeter (PE, USA).

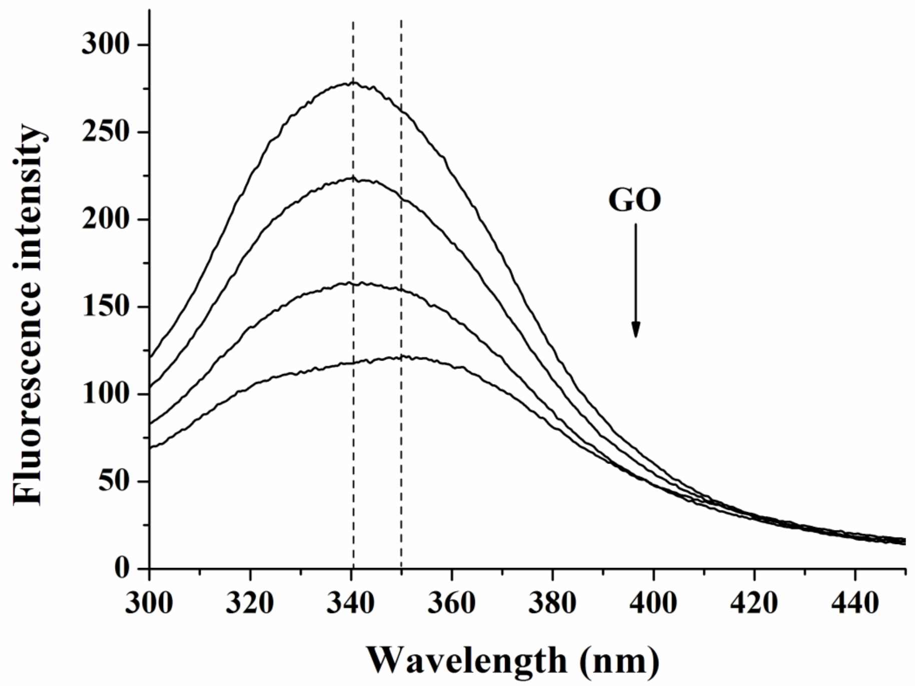

Figure S1 Fluorescence quenching of the Taq polymerase in the presence of GO. Polymerase: 1 μL (5000 units/mL); Concentration of GO (From up to down): 0, 1, 2, 4 μg/mL; pH=7.4; ex=280 nm.

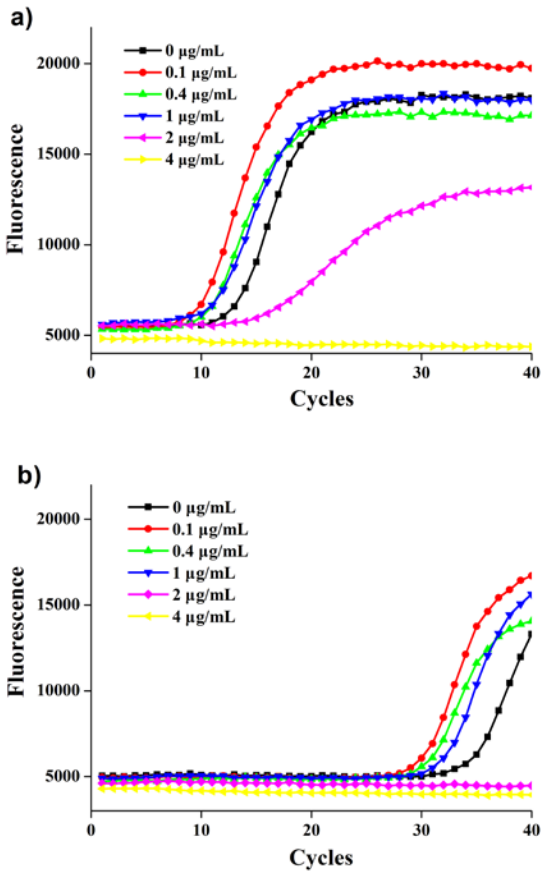

Figure S2 Real-time PCR curves by plotting SYBR Green I fluorescence intensity versus cycle number. The involved concentrations of GO in the samples were varied as labeled for each curve. **a**) Matched R1 as reverse primer only; **b**) mismatched W1 as reverse primer only. The concentrations of Taq polymerase, primers and the template DNA were kept constant.

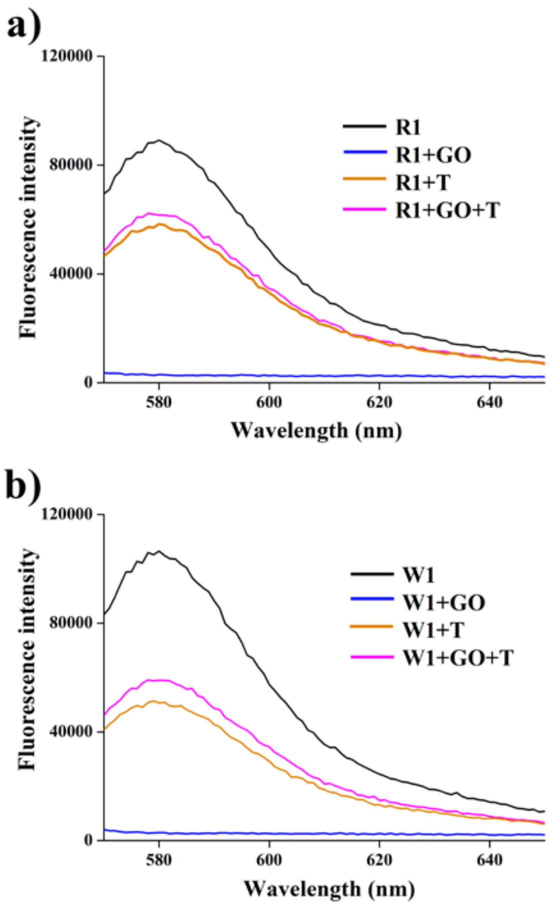

Figure S3 Fluorescence emission spectra of 10 nM TAMRA labelled R1 (**a**) and W1 (**b**) to sequential addition of GO (20 µg/mL) and template DNA (100 nM).

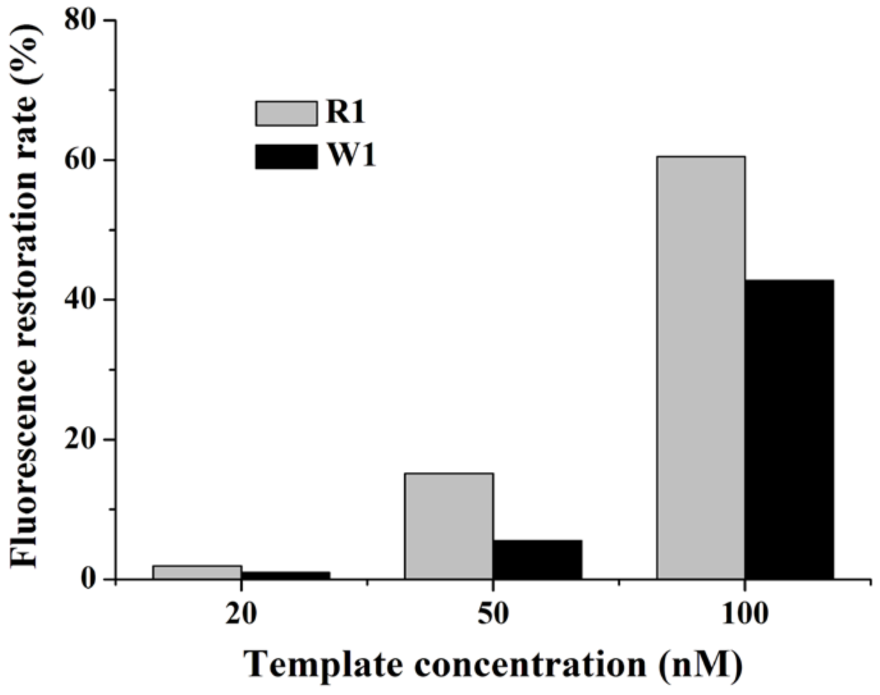

Figure S4 Comparison of fluorescence restoration rate of TAMRA labelled R1 and W1 by addition of GO (20 μg/mL) and template DNA (20, 50, and 100 nM).

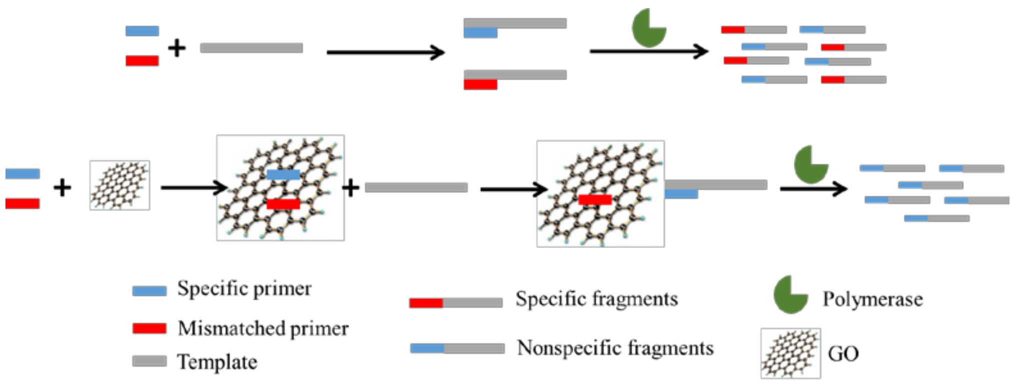

Figure S5 Schematic mechanism of PCR specificity enhanced by GO。
